# Seroprevalence of Hepatitis C Viral Infection in Ethiopia: A Systematic Review and Meta-Analysis

**DOI:** 10.1155/2021/8873389

**Published:** 2021-04-09

**Authors:** Teshiwal Deress, Yihenew Million, Teshome Belachew, Mohabaw Jemal, Mekonnen Girma

**Affiliations:** ^1^Unit of Quality Assurance and Laboratory Management, School of Biomedical and Laboratory Sciences, College of Medicine and Health Sciences, University of Gondar, Gondar, Ethiopia; ^2^Department of Medical Microbiology, School of Biomedical and Laboratory Sciences, College of Medicine and Health Sciences, University of Gondar, Gondar, Ethiopia

## Abstract

**Background:**

Hepatitis C virus is a highly genetically heterogenous bloodborne pathogen that is responsible for acute and chronic hepatitis. Globally, an estimated 71 million population is chronically infected with this virus from which 399,000 people die every year. Its prevalence is high in Ethiopia and varies from region to region, even among different studies within a region.

**Methods:**

Electronic databases, including Science Direct, Medline, HINARI, African Journals Online, TRIP database, African Index Medicus, and Directory of Open Access Journals, searched from 2010 to 2020 and published articles were included. Due to evidence of considerable heterogeneity, the pooled prevalence of anti-HCV was analyzed using the random-effects model. The possible sources of heterogeneity were analyzed through subgroup analysis, sensitivity analysis, and meta-regression. Funnel plots and Egger's test statistics were used to determine the presence of publication bias.

**Results:**

The analysis of 56 articles showed that the prevalence of anti-HCV in Ethiopia ranged from 0% to 22%. The pooled prevalence estimated was 2% (95% CI 2.0–3.0), and the meta-regression statistics indicated that the diagnostic method (*p*=0.037), study group (*p*=0.005), and level of bias (*p*=0.035) showed statistically significant association with the outcome variable. The sensitivity analysis claims no influence on the overall effect estimate while removing a single study from the analysis at a time. Egger's test statistics (*p* ≤ 0.001) declare the presence of publication bias that is handled using time and fill analysis.

**Conclusions:**

The pooled prevalence of anti-HCV in Ethiopia was high. Predictor variables, including the diagnostic method, study group, and level of bias, showed a statistically significant relationship with the outcome variable. Strengthening the scope of existing prevention and control programs and implementing novel approaches, including screen-and-treat, could significantly help to tackle this critical public health issue. The study provides a current estimate which is valuable for policymakers and other responsible bodies.

## 1. Background

Hepatitis C virus (HCV) is an enveloped positive-sense single-stranded RNA molecule of approximately 9500 nucleotides, which is grouped under the genus Hepacivirus [1]. It is genetically highly heterogenous, which is classified into seven genotypes (1–7) with approximately a hundred subtypes [2]. The virus is a bloodborne pathogen that is commonly transmitted through direct blood contact, mother to child, organ transplantations, inadequate sterilization of medical equipment, unsafe sexual practices, and intravenous drug use [3–5].

Hepatitis C virus is responsible for acute and chronic hepatitis [6]. It is a significant public health issue because of its chronic hepatitis that often progresses to cirrhosis and hepatocellular carcinoma [1]. The acute infection is usually an asymptomatic stage. Among HCV-infected patients, the viral particle gradually decreases in 15–25% of patients and finally disappears from the blood circulation. Though the rate of progression to chronic infection is affected by several factors, usually, on average, 75–85% of patients will progress to chronic disease [7]. The persistent HCV infection is typically related to the development of liver cirrhosis and hepatocellular carcinoma [8]. The severity of the infection is mainly due to its long-term hepatic and extrahepatic consequences [9]. Within twenty years of disease progression, 27% and 25% of the patients will develop liver cirrhosis and hepatocellular carcinoma, respectively [7]. The most frequent complaint in chronic HCV infection is fatigue, and other less common clinical manifestations are anorexia, weakness, nausea, arthralgia, myalgia, and weight loss [10]. The primary prevention mechanisms are essential in reducing the risks of exposure through education on safe sex, safe protocols of contaminated needle use, and blood and other body fluids [11]. Regarding diagnostic techniques, the initial screening test is the antibody test. Currently, different testing methods are available on the market, including enzyme-linked immunosorbent assay (ELISA), reverse transcription polymerase chain reaction (RT-PCR), and rapid diagnostic test (RDT) kits [12].

Hepatitis C virus causes substantial morbidity and mortality worldwide [2, 13]. An estimated 71 million population is chronically infected with the virus, and about 399,000 people die each year due to cirrhosis and liver cancer globally [14, 15]. According to the World Health Organization (WHO) estimation, during 2015, there was 1.75 million population with new HCV cases globally [5]. The virus is about four times more infectious than the human immunodeficiency virus (HIV) [16]. The lack of an effective vaccination can significantly increase its burden [5]. Besides, the vast genetic diversity brings challenges to the host immune control, development of pangenotypic treatments, and patient management [17]. Hepatitis C virus infection is also a significant public health issue in Ethiopia, with its prevalence estimate ranging from <0.5% [18–24] to 22% [25]. Even the prevalence varies from study to study within a region depending on the nature of the study participants, study setting, year of study, and other potential predictor variables. The first meta-analysis and systematic review were conducted in 2016 [26]; however, due to increased demand for current prevalence data by the health regulatory bodies to design the appropriate intervention strategies, conducting the current pooled prevalence estimate of the anti-HCV was found to be useful. Therefore, this systematic review and meta-analysis aimed to generate the pooled prevalence estimate of the anti-HCV by including a substantial number of articles published from 2010 to December 20, 2020, among the Ethiopian population.

## 2. Methods

### 2.1. Study Design and Protocol Registration

The protocol of the current systematic review and meta-analysis was designed following the “Preferred Reporting Items for Systematic Reviews and Meta-Analysis Protocols” (PRISMA-P 2015) guidelines [27] and prospectively registered in the PROSPERO database with the protocol registration number of CRD42020153487.

### 2.2. Article Searching Strategy

Before starting the actual work of the project, the Database of Abstracts of Reviews of Effects (DARE) (http://www.library.UCSF.edu) and PROSPERO databases regressively searched to check the presence of similar projects related to the topic. The literature searching strategy, selection of potentially eligible studies, data extraction, data analysis, and result reporting were performed according to the Preferred Reporting Items for Systematic Reviews and Meta-Analyses (PRISMA) guidelines [28]. Electronic databases, namely, Science Direct, Medline through PubMed, HINARI, African Journals Online (AJOL), TRIP database, African Index Medicus, and Directory of Open Access Journals (DOAJ), have been mined using a combination of keywords and Boolean operators. Keywords including hepatitis C virus, hepatitis C, HCV, viral liver disease, viral hepatitis, hepacivirus, hepatitis C antibodies, transfusion-transmissible infectious, prevalence, seroprevalence, epidemiology, seroepidemiology, proportion, rate, frequency, magnitude, Ethiopia, and the year of publication were used to search the mentioned databases. According to the requirements of the database, the search strings were designed with the help of librarian experts. Our Medline searching strategy is provided in Supplementary Material. Besides, to include as many articles as possible, manual hand searching on Google, Google Scholar, and screening of reference lists of both included and excluded studies were performed. All studies published from 2010 to 2020 were considered, and the most recent database search was performed on December 20, 2020.

### 2.3. Article Selection and Eligibility

The searched articles were imported into the EndNote X9 software, and duplicate studies were removed. All authors screened the remaining records independently by title, abstract, and full-text to identify potentially eligible studies for the review. Studies were eligible if they were primary study full-text articles conducted in Ethiopia and published in peer-reviewed journals from 2010 to 2020 in the English language. Besides, studies conducted with anti-HCV laboratory screening, prevalence data clearly stated, or if missed the presence of sufficient data to calculate the prevalence (known sample size and anti-HCV positive finding) were considered. Regarding the exclusion criteria, studies with zero and unclear prevalence and those having methodological errors were excluded from the study.

### 2.4. Data Extraction

The data abstraction form was prepared in a Microsoft Excel Spreadsheet which includes first author's name, year of study, publication year, region, setting (urban or rural), the HCV diagnostic method (ELISA or RDT), the study group, study design, sample size, sampling technique, and the number of anti-HCV positive cases. Two (TD and YM) authors extracted the data independently. A third author (TB) confirmed the data extraction process by taking five studies randomly and any inconsistency resolved by mutual consensus.

### 2.5. Quality Assessment

The quality assessment was performed independently by two authors using the Joanna Briggs Institute (JBI) quality assessment tool for prevalence studies [29]. The instrument is composed of 9 quality domains where each item scored either positive or negative, and the importance of the items was not weighted. Higher scores (positive items) correspond to higher-quality studies for our review. We considered that the studies with scores 0–3, 4–6, and 7–9 represented a high, moderate, and low risk of bias, respectively. The quality assessment score did not use for study selection for the present review. This variable (score in the quality evaluation of the study) was analyzed in meta-regression. The quality of data abstraction (interrater agreement) examined using Cohen's Kappa and the reliability coefficient (Kappa value) was found to be 0.827 (*p* ≤ 0.001), which indicates an excellent agreement.

### 2.6. Data Synthesis and Analysis

Data were analyzed using Metaprop package of Stata software which is a statistical program used to perform meta-analyses of proportions in Stata. During the analysis, the Freeman Tukey double arcsine transformation (ftt) was enabled to include proportions close to 0 and 1 [30]. This program computes the weighted pooled estimate and then performs back-transformation on the pooled estimate. The time-transformed prevalence weighted very slightly towards 50%, which enable 0 prevalence studies included in the analysis [31]. When there is evidence of a cross-study heterogeneity, the random-effects model is recommended for analysis [32]. In this case, the DerSimonian and Laird method is most commonly used [33]. The presence of heterogeneity among studies is checked using *I*^2^ test statistics, which estimates the presence of observed differences between studies due to heterogeneity. The *I*^2^ value can range from 0 to 100%, and 0% indicates the absence of heterogeneity, whereas 100% is a definitive indicator of significant heterogeneity. The 25%, 50%, and 75% values represent low, medium, and high heterogeneities between studies, respectively [34]. Besides, a *p* value of <0.05 is used to declare the presence of heterogeneity [35]. In this meta-analysis, the *I*^2^ value was high (97.77%), which is >75%, an indication of considerable heterogeneity. Due to this reason, the analysis conducted using a random-effects model at 95% CI as opposed to the fixed effects model to adjust the observed variability among studies. The possible sources of heterogeneity are investigated through subgroup analysis, sensitivity analysis, and meta-regression. The visual inspection of funnel plots and Egger's weighted statistics were used to investigate the presence of publication bias and small study effects. All the data management and statistical analysis performed using Stata software version 16.0 (StataCorp LLC College Station TX 77845, USA for windows version).

## 3. Results

### 3.1. Study Selection

**Figure 1:**
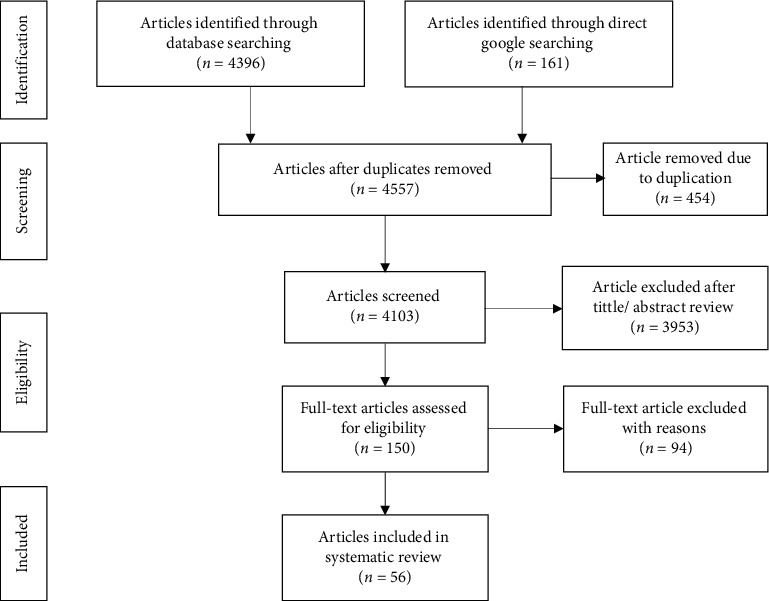
PRISMA flow diagram for identification and selection of articles for inclusion in the review.

Initially, 4557 studies were retrieved from the databases and hand searching. From this, 454 studies were removed due to duplication. Then, 4103 articles were screened by title/abstract, and 3953 articles were excluded because of no relevance to the current review. The remaining 150 full-text articles were further refined, and 94 of them were excluded due to being review articles, zero prevalence, articles published in nonreputable (nonpeer-reviewed) journals, and studies conducted before 2010. Finally, 56 studies [18–25, 36–83] fulfilled the inclusion criteria and were included in the review (Figure 1).

### 3.2. Overview of Anti-HCV Prevalence Studies

Hepatitis C virus prevalence data and other indicators were extracted from 56 studies. The overall study participants used for the HCV screening were 710820, which was obtained from six regional states and two self-administrative cities (SAC) of the country. Studies with the smallest and largest sample sizes were 120 from the Addis Ababa city [25] and 554954 from all regions of the country [77]. All included studies were conducted with cross-sectional study designs, and the most recent studies were conducted in 2020 [77, 79, 83]. Concerning HCV study coverage in the country, the highest number of studies, 25 (44.64%), was obtained from the Amhara region, followed by 9 (16.07%) from the Southern Nations Nationalities and Peoples Regions (SNNPR). A minimal number of studies were obtained from Tigray, Gambella, Somali, and Harari regions; however, no study was obtained from Benishangul-Gumuz and Afar regions (Table 1).

**Figure 2:**
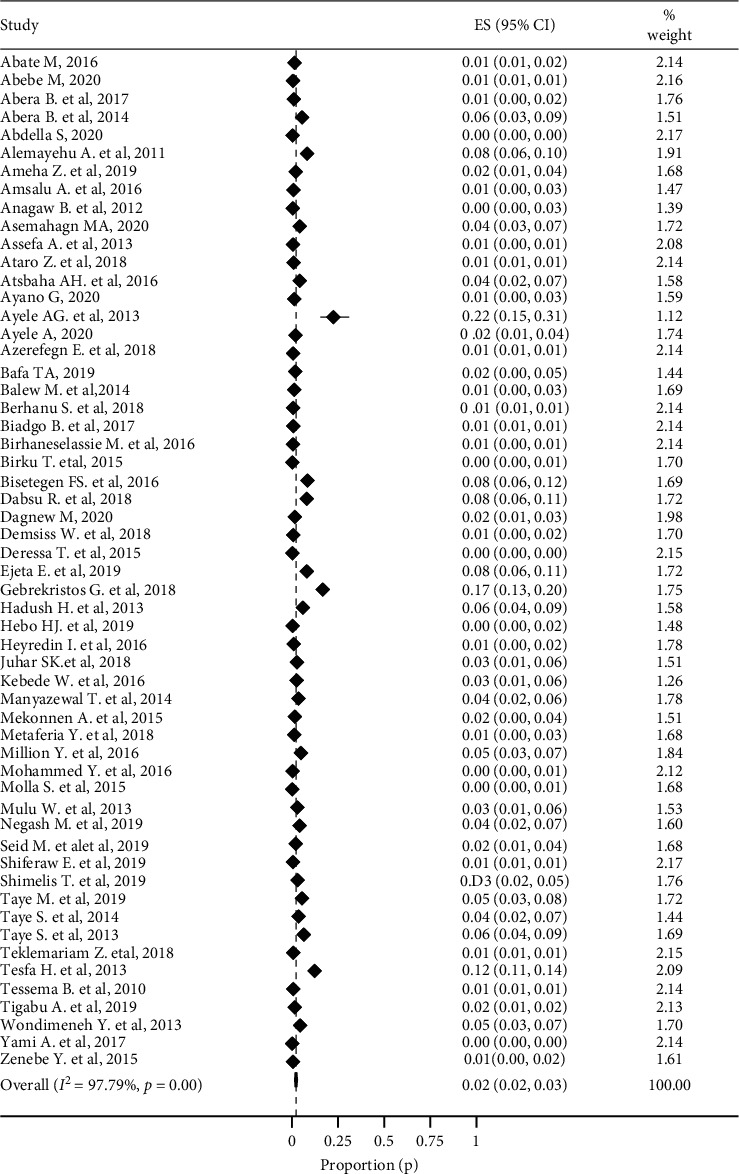
Poole prevalence of anti-HCV in Ethiopia from 2010 to 2020.

There was high variability in the prevalence of anti-HCV among primary studies in Ethiopia, which can range from 0% [18–24, 84] to 22% [25]. Due to the presence of considerable heterogeneity (*I*^2^ = 97.79) among the included studies, the random-effects model was used to estimate the pooled prevalence. According to the random-effects model, the pooled prevalence estimate of anti-HCV was 2% (95% CI: 2-3) with a *p* value of ≤0.01 (Figure 2).

### 3.3. Investigation of Heterogeneity

#### 3.3.1. Subgroup Analysis

A subgroup analysis was performed on the study group, risk of bias, region/providence, year of publication, sampling technique, and diagnostic method. The subgroup analysis showed that the heterogeneity level slightly reduced among studies conducted on HIV-positive study participants; while in all cases, the level of heterogeneity was still high. Concerning prevalence estimates, the highest anti-HCV prevalence reported among HIV patients (4%), studies with a high risk of bias (5%), nonprobability sampling techniques (7%), studies conducted in SAC (5%), and RDT diagnostic methods (3%) than estimates of the corresponding subgroups (Table 2).

#### 3.3.2. Meta-Regression and Sensitivity Analysis

A meta-regression analysis was performed on categorical variables, including publication year, the study group, region, sample size, sampling technique, risk of bias, and diagnostic methods. Among these predictor variables, the region was marginal (*p*=0.089); whereas, the diagnostic method (*p*=0.037), study group (*p*=0.005), and level of bias (*p*=0.035) showed a statistically significant association with the outcome variable. Furthermore, a sensitivity analysis was conducted to examine the influence of a single study on the overall effect estimate while removing a study at a time from the analysis; however, the pooled estimate did not significantly change (Table 3).

#### 3.3.3. Publication Bias and Small Study Effects

**Figure 3:**
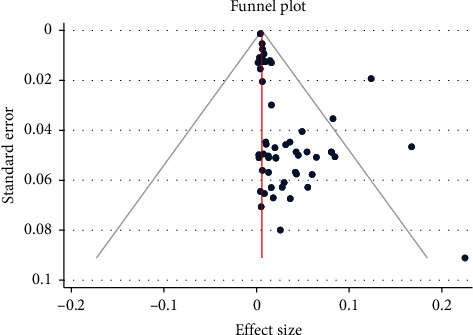
Funnel plot of anti-HCV prevalence in Ethiopia published from 2010 to 2020.

Publication bias was assessed through visual inspection of the funnel plot and objectively using Egger's weighted regression statistics. Each dot in the funnel plot represented a single study, and the symmetrical distribution suggests the absence of publication bias [85]. Studies' effect sizes were plotted against the corresponding standard errors, and the visual inspection of the funnel plot showed the presence of publication bias (Figure 3).

**Figure 4:**
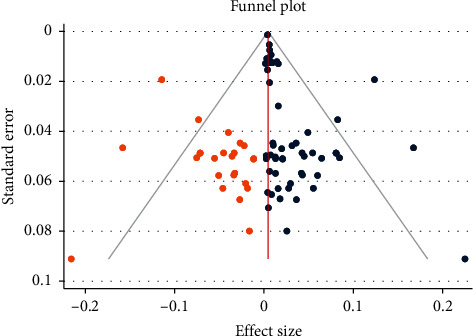
Trim and fill analysis of anti-HCV prevalence studies in Ethiopia published from 2010 to 2020.

Then, Egger's test result declared the presence of publication bias (*p* ≤ 0.001). Finally, the funnel plot was adjusted using the trim and fill analysis (Figure 4).

## 4. Discussion

Hepatitis C virus is a global threat that mainly affects developing countries where there is an inadequate infrastructure for prevention and control [86]. So far, studies indicated that the highest prevalence rates of HCV were reported from sub-Saharan Africa and Asia [87]. As a part of developing countries, HCV prevalence is high in Ethiopia and variable from region to region; even, it differs among studies conducted within an area. For this reason, generating pooled prevalence and providing detailed analysis and explanation could significantly help policymakers and other stakeholders in designing the proper strategy for intervention. The analysis of 56 full-text articles showed that the prevalence of anti-HCV ranged from 0% to 22%. From this, the pooled prevalence estimate was 2%, which is nearly similar to 1.9%, 2.5%, and 2.9% of findings obtained from Yemen, Sudan, and Congo [88–90]. The current result was lower than the earlier estimate (3.1%) conducted in the country [26], which indicates a slight decline in prevalence in the country. Probably, the decrease in the prevalence could be due to the attention given by the Federal Ministry of Health, health workers, and other responsible bodies for infection prevention and control. The result was, however, much lower than 3.4%, 4.8%, 6.2%, 6.5%, and 11.9% findings obtained from Africa, Somalia, Pakistan, Cameroon, and Egypt [91–94]. On the other hand, the current pooled estimate is far higher than (0.9% and 1%), 0.3%, 0.91%, and 1% findings obtained from the eastern parts of Africa, Iran, China, and the global pooled estimate [15, 87, 88, 95]. The prevalence difference among the mentioned countries could be due to differences in health programs, diagnostic methods, and sociocultural practice contributed to disease transmission. Mainly, in Ethiopia, community practices such as tattooing and medical injections administered by other than health professionals are widely practiced by the Ethiopian community.

Out of fifty-six studies, six reported the prevalence of anti-HCV among HIV-positive patients. It is a fact that HIV infection potentially affects the natural history of HCV infection. Findings indicated that nearly one-third of HIV-uninfected persons spontaneously clear HCV infection in the first year; however, HCV coinfection with HIV significantly reduces the clearance rate of HCV viral particles from the blood circulation. In the current review, the prevalence estimate of anti-HCV among HIV-positive patients was 4%, which is similar to the global pooled estimate of 2.4% [96]. This high prevalence could be attributed due to the high burden of coinfection in the area, although the result showed a considerable decline compared to the previous estimate (5.5%) [26]. The decrease in the prevalence could be due to factors including mortality of the infected population, reduction of new cases because of the implementation of blood supply screening, and decline of high-risk behaviors among the community. On the other hand, quite higher findings were obtained from Cameroon (7.13%), sub-Saharan regions (7%), and Iran (10.95%) [94, 97, 98]. The 2% pooled estimate among the pregnant women is quite similar to findings from Congo (3.3%) and Cameroon (3%), but higher than a study from Sudan (0.6%) [89, 90, 94]. The result was, however, much lower than the pooled global estimate (6.4%) and a finding from Egypt (9%) [96, 99].

In Ethiopia, infectious diseases that can be transmitted through blood transfusion have been prevented by excluding blood donors having specific risk factors, using healthy donors, screening donated blood with reliable screening techniques, and maintaining good hygienic practices. Despite several interventions implemented so far, HCV is still a significant public health issue in the country. The insignificant decline of the prevalence could be due to the nature of the infection (chronic infection) that has the potential to transmit the disease to the healthy person and the lack of an effective vaccine to prevent the incidence of new cases. Considering the vast population of the country, a prevalence of 1% (our result) leads to thousands and even hundreds of thousands of seropositive patients. In the current study, the prevalence of viral markers among blood donors is much lower than the general population; this could be due to donors selected from populations with the low risk of infectious diseases due to donor health assessment. This result is far lower than findings from Congo (2.7%), Cameroon (2.49%), Egypt (10.4%), and China (8.68%) [90, 94, 99, 100]; however, it is higher than 0.5% a finding from Iran [101]. According to study quality, the highest prevalence of anti-HCV (5%) was noted among studies that have a high risk of bias in their method compared to low risk of bias studies (2%). The current estimate showed a significant difference to the diagnostic method in anti-HCV prevalence, and studies conducted using rapid diagnostic test kits showed a higher estimate (3%) than studies conducted using ELISA (2%). This difference could be since rapid diagnostic tests might lack specificity compared to ELISA tests and wrongly increased the prevalence. Hence, studies conducted with molecular techniques alone stand for the actual prevalence estimate.

According to the meta-regression analysis, the prevalence of anti-HCV in Ethiopia has significantly reduced as the studies used ELISA instead of a rapid test for the diagnosis of anti-HCV.

Regarding the risk of bias, when the bias level of the included studies increases, the prevalence estimate substantially increases. Besides, study groups of the articles included had a statistically significant relationship with the pooled prevalence estimate.

This review article incurred several limitations that should be acknowledged. The first limitation is the lack of including unpublished studies. Among the included studies, nine of them had a high level of bias. Besides, a substantial number of studies were undertaken on blood donors, which potentially underestimate the national anti-HCV pooled prevalence. The other drawback is the presence of considerable statistical heterogeneity among the included studies. Besides, there were limited studies obtained from several regions that could compromise the representativeness of the pooled estimate.

## 5. Conclusions

Even though several approaches increase diagnosis, treatment, and prevention through health education for the last decade, the result of this review showed that HCV is still a significant public health issue in Ethiopia. Studies that used rapid tests instead of ELISA (golden method) for the diagnosis of anti-HCV positively affected the pooled estimate. Besides, the study group and level of bias showed a relationship with the pooled prevalence estimate. Strengthening the scope of existing prevention and control programs is recommended. Besides, implementing novel approaches, including screen-and-treat, could significantly help to reduce the burden of HCV disease in Ethiopia. Further political will and strong community awareness could be critical to effectively tackling the burden of HCV. This study has a serious limitation, since many included studies have extremely low prevalences and extremely narrow confidence intervals; we are aware that in similar circumstances, the power of the meta-analysis is low and interpretation of the results may be misleading.

## Figures and Tables

**Table 1:** Characteristics of the included studies in the systematic review and meta-analysis for the prevalence of anti-HCV in Ethiopia, 2020.

First author, publication year [reference]	Region	Study group	Sampling technique	Sample	Positive	Diagnostic method	Risk of bias
Abate and Wolde, 2016 [36]	Somali	Blood donors	Consecutive	6827	99	ELISA	Moderate
Abebe et al., 2020 [78]	Oromia	Blood donors	Consecutive	17810	114	ELISA	
Abera et al., 2017 [37]	Amhara	Adult population	Random	481	5	RDT	High
Abera et al., 2014 [38]	Amhara	HIV	Random	253	14	ELISA	Moderate
Abdella et al., 2020 [77]	Nation-wide	Blood donors	Random	554954	2220	ELISA	
Alemayehu et al., 2011 [39]	SNNPR	Mixed	Consecutive	800	66	ELISA	Moderate
Ameha et al., 2019 [76]	Amhara	VCT	Random	382	8	RDT	Moderate
Amsalu et al., 2016 [40]	SNNPR	Mixed	Entire	234	2	RDT	High
Anagaw et al., 2012 [18]	Amhara	Mixed	Not stated	200	1	RDT	High
Asemahagn, 2020 [79]	Amhara	Surgical	Random	422	18	RDT	
Assefa et al., 2013 [41]	Amhara	Blood donors	Entire	2384	15	ELISA	Moderate
Ataro et al., 2018 [42]	SAC	Blood donors	Entire	6376	61	ELISA	Low
Atsbaha et al., 2016 [43]	Tigray	Mixed	Random	302	13	RDT	Low
Ayano et al., 2020 [80]	SAC	Psychiatric	Random	309	4	ELISA	
Ayele and Gebre-Selassie, 2013 [25]	SAC	Liver disease	Convenient	120	27	RDT	High
Ayele et al., 2020 [81]	Gambella	Refugees	Convenient	453	9	ELISA	
Azerefegn et al., 2018 [73]	SNNPR	Blood donors	Entire	6849	48	ELISA	Low
Bafa and Egata, 2019 [82]	SNNPR	Pregnant women	Random	222	4	ELISA	
Balew et al., 2014 [44]	Amhara	HIV	Random	395	5	RDT	Low
Berhanu S, 2018 [74]	Amhara	Blood donors	Entire	7255	49	ELISA	Moderate
Biadgo et al., 2017 [45]	Amhara	Blood donors	Entire	6471	51	ELISA	Low
Birhaneselassie et al., 2016 [46]	SNNPR	Blood donors	Entire	6337	38	ELISA	Moderate
Birku T, 2015 [19]	Amhara	Military personnel	Random	403	1	RDT	Low
Bisetegen et al., 2016 [47]	SNNPR	Blood donors	Consecutive	390	33	ELISA	Moderate
Dabsu Ejeta, 2018 [48]	Oromia	Pregnant women	Convenient	421	34	RDT	Low
Dagnew M, 2020 [83]	Amhara	Pregnant women	Random	1121	18	ELISA	
Demsiss et al., 2018 [49]	Amhara	Health students	Random	408	3	ELISA	Low
Deressa et al., 2018 [20]	Amhara	Blood donors	Entire	8460	27	ELISA	Low
Ejeta and Dabsu, 2019 [50]	Oromia	Pregnant women	Consecutive	421	34	ELISA	Low
Gebrekristos et al., 2018 [51]	Tigray	Mixed	Not stated	460	77	ELISA	High
Hadush et al., 2013 [52]	Tigray	Mixed	Not stated	300	18	ELISA	High
Hebo et al., 2019 [21]	Oromia	Health workers	Random	240	1	ELISA	Low
Heyredin et al., 2019 [53]	Mixed	Blood donors	Consecutive	500	5	ELISA	Low
Juhar et al., 2018 [54]	SAC	Hemodialysis	Not stated	253	7	ELISA	High
Kebede et al., 2017 [55]	Oromia	Prisoner	Random	156	4	ELISA	Moderate
Manyazewal et al., 2014 [56]	SAC	Mixed	Not stated	500	18	ELISA	Low
Mekonnen et al., 2015 [57]	SAC	Waste handlers	Random	252	4	ELISA	Low
Metaferia et al., 2018 [58]	Amhara	Pregnant women	Consecutive	385	5	RDT	Low
Million et al., 2019 [75]	Amhara	Mixed	Convenient	610	30	ELISA	Low
Mohammed and Bekele, 2016 [22]	Somali	Blood donors	Entire	4224	17	ELISA	Moderate
Molla et al., 2015 [23]	Amhara	Pregnant women	Random	384	1	RDT	Low
Mulu et al., 2013 [59]	Amhara	HIV	Not stated	269	8	ELISA	High
Negash et al., 2019 [60]	Amhara	Blood donors	Consecutive	310	13	ELISA	Low
Seid et al., 2014 [61]	Amhara	Pregnant women	Random	385	8	RDT	Low
Shiferaw et al., 2019 [62]	Amhara	Blood donors	Entire	35435	213	ELISA	Low
Shimelis et al., 2019 [63]	SNNPR	HIV	Not stated	477	15	RDT	Low
Taye et al., 2019 [64]	SNNPR	Surgery patients	Random	422	23	RDT	Moderate
Taye et al., 2014 [65]	SNNPR	Chronic hepatitis	Not stated	220	8	RDT	High
Taye and Lakew, 2013 [66]	SAC	HIV	Nonprobability	387	25	RDT	Moderate
Teklemariam et al., 2018 [67]	Harari	Blood donors	Entire	11382	91	ELISA	Low
Tesfa et al., 2013 [68]	Amhara	General	Entire	2684	332	RDT	Moderate
Tessema et al., 2010 [69]	Amhara	Blood donors	Consecutive	6361	45	ELISA	Low
Tigabu et al., 2019 [70]	Amhara	Blood donors	Entire	5983	96	ELISA	Low
Wondimeneh et al., 2013 [71]	Amhara	HIV	Not stated	400	18	RDT	Low
Yami et al., 2011 [24]	Oromia	Blood donors	Random	6063	10	ELISA	Low
Zenebe et al., 2015 [72]	Amhara	Pregnant women	Nonprobability	318	2	ELISA	Moderate

SAC, self-administrative cities (Addis Ababa and/or Dire Dawa); ELISA, enzyme-linked immunosorbent assay; RDT, rapid diagnostic test; SNNPR, Southern Nations, Nationalities and Peoples Region; HIV, human immune virus; VCT, voluntary counseling and testing.

**Table 2:** Subgroup analysis of the anti-HCV pooled prevalence estimation in Ethiopia, 2020.

Predictor variables	Variable category	Included studies	ES (95% CI)	*I* ^2^ %	*p* value
Study group	Blood donor	19	0.01 (0.01, 0.01)	95.96	≤0.001
	HIV positive	6	0.04 (0.02, 0.06)	74.13	≤0.001
	Pregnant women	8	0.02 (0.01, 0.05)	91.83	≤0.001
	Mixed groups	8	0.05 (0.02, 0.08)	93.76	≤0.001
	Others	15	0.03 (0.01, 0.06)	96.48	≤0.001

Risk of bias	Low	32	0.02 (0.01, 0.02)	95.88	≤0.001
	Moderate	16	0.03 (0.01, 0.04)	98.56	≤0.001
	High	9	0.05 (0.02, 0.09)	95.30	≤0.001

Region/city	Amhara	25	0.02 (0.01, 0.02)	97.67	≤0.001
	Oromia	6	0.02 (0.01, 0.04)	97.36	≤0.001
	SNNPR	9	0.03 (0.01, 0.05)	96.79	≤0.001
	Self-administrative city	7	0.05 (0.02, 0.09)	95.97	≤0.001
	Others	9	0.02 (0.01, 0.03)	97.96	≤0.001

Year of publication	2010–2012	3	0.02 (0.00, 0.09)	—	—
	2013–2015	16	0.03 (0.01, 0.06)	98.39	≤0.001
	2016–2020	37	0.02 (0.01, 0.02)	96.75	≤0.001

Sampling techniques	Probability	19	0.02 (0.01, 0.02)	92.22	≤0.001
	Entire sampling	23	0.02 (0.01, 0.02)	98.19	≤0.001
	Nonprobability	7	0.02 (0.02, 0.09)	97.55	≤0.001
	Not stated	7	0.05 (0.02, 0.09)	93.65	≤0.001

Diagnostic method	ELISA	37	0.02 (0.01, 0.02)	96.88	≤0.001
	RDT	19	0.03 (0.02, 0.06)	95.90	≤0.001

ELISA, enzyme-linked immunosorbent assay; RDT, rapid diagnostic test; SNNPR, Southern Nations, Nationalities and Peoples Region.

**Table 3:** Meta-regression analysis of factors for the heterogeneity of anti-HCV prevalence in Ethiopia, 2020.

Predictor	Coefficient	Std. error	*p* value	Adjusted *R*^2^ (%)
Sample size	−3.30*e* − 06	2.04*e* − 06	0.112	−0.58
Publication year	−0.0693	0.0605	0.257	1.34
Region	0.1790	0.1034	0.089	0.39
Study group	0.2916	0.0989	0.005^*∗*^	17.66
Diagnostic method	0.7042	0.3284	0.037^*∗*^	−9.26
Level bias	0.4482	0.2066	0.035^*∗*^	9.04

^*∗*^Statistically significant association.

## Data Availability

The data generated or analyzed during this study are included within the article.

## Abbreviations

AJOL: African Journals Online
DARE: Database of Abstracts of Reviews of Effects
ELISA: Enzyme-linked immunosorbent assay
HCV: Hepatitis C virus
HIV: Human immunodeficiency virus
JBI: Joanna Briggs Institute
PRISMA: Preferred Reporting Items for Systematic Reviews and Meta-Analyses
RDT: Rapid diagnostic tests
RT-PCR: Reverse transcription polymerase chain reaction
SNNPR: Southern Nations, Nationalities and Peoples Region
VCT: Volunteer for counseling and testing
WHO: World Health Organization.

## Supplementary Materials

Supplementary Materials Medline search strategy.
