# Supplementary material for: Seroprevalence of Hepatitis C Viral Infection in Ethiopia: A Systematic Review and Meta-Analysis
Source: ScientificWorldJournal. 2021 Apr 9;2021:8873389. doi: 10.1155/2021/8873389 (PMC8052182; doi:10.1155/2021/8873389)
Supplement: Supplementary Materials — Medline search strategy. [file 8873389.f1.docx]

**Medline Search Strategy**

[(“hepatitis C virus” OR “hepatitis C” OR “hepatitis C infection” OR hepacivirus OR “hepatitis c antibodies” OR HCV OR “viral liver disease” OR “transfusion-transmissible infection” OR “viral hepatitis”) AND (prevalence OR sero-prevalence OR frequency OR epidemiology OR sero-epidemiology OR magnitude OR proportion OR rate) AND Ethiopia AND year]
